# Helicobacter pylori Infection in Persons with Intellectual Disability in Residential Care in Israel

**DOI:** 10.1100/tsw.2001.61

**Published:** 2001-07-17

**Authors:** Joav Merrick, Shoshana Aspler, Inessa Dubman

**Affiliations:** National Institute of Child Health and Human Development, Division for Mental Retardation, Ministry of Labour and Social Affairs, Jerusalem, Israel

**Keywords:** Helicobacter pylori infection, dyspepsia, institutionalized persons, residential care, mental retardation, developmental disability, intellectual disability, Israel

## Abstract

Helicobacter pylori (formerly Campylobacter pylori) was identified in 1982 by researchers from Australia as a pathogenic factor in peptic ulcer disease. Due to the few studies on H. pylori infection conducted in the population of persons with intellectual disability it was decided to conduct a clinical study in Israel. The purpose of the study was to determine the occurrence of H. pylori infection in persons who presented with severe dyspeptic symptoms and to monitor clinically the effect of treatment. The Division for Mental Retardation in Israel provides service to 6,022 persons in 53 residential care centers and 1 in central Israel was selected for this pilot study. The study has been performed since 1999 and each patient who came to the medical clinic of the institution with severe dyspeptic symptoms was examined clinically and a blood specimen drawn for IgG antibodies to H. pylori (ELISA, Pharmatop Millenia). In case of positive serology, triple drug treatment (amoxycillin, metronidazole, and pantoprazole or omepra-zole) was initiated for 1 week. Since 1999 a total of 43 persons (total population in care was 224) had severe dyspeptic symptoms and 42 persons (98%, 26 males, 16 females, mean age 45 years, mean institutionalization 20 years) had Helicobacter infection. All patients were treated for 1 week, but six patients received an extra month of omeprazole due to persistent symptoms. At follow-up, clinically all patients had improvement and only seven still had minor complaints (83% treatment success). Persons with developmental disability, intellectual disability, or mental retardation in residential care presenting with severe dyspeptic symptoms had a high incidence of H. pylori infection. Therefore, we recommend serology or urea breath investigations in this population presenting with dyspeptic symptoms and triple drug treatment for 1 week in case of positive findings.

## INTRODUCTION

Peptic ulcer disease, defined as a chronic inflammation of the stomach and duodenum, has been thought of as a disorder associated with stress and dietary factors, which resulted in bed rest treatment and special diets. Later the concept of gastric acid secretions was introduced and antacids became a standard therapy. In 1971, Sir James Black identified a subtype of the histamine receptor ($H_2$-receptor) that appeared to be the principal mediator of gastric acid secretion and antagonists of this receptor were introduced[1].

Inhibitors of the proton pump in the gastric parietal cells, bismuth compounds, and prostaglandins have also been developed as anti-ulcer drugs, but the problem is still a high recurrence rate of peptic ulcers, even after complete healing of the ulcer[1].

In 1982 another important pathogenic factor in peptic ulcer disease was identified when J. Robin Warren and Barry J. Marshall[2] from Royal Perth Hospital in Australia discovered a spiral, Gram-negative flagellate and microaerophilic bacteria in the narrow interface between the gastric epithelial cell surface and the overlying mucus gel in biopsies from patients with active chronic gastritis, duodenal ulcer or gastric ulcer. This bacteria was named *Campylobacter pylori*, but in 1989 renamed *Helicobacter pylori*[3].

*Helicobacter pylori* (*H. pylori*) causes gastritis and ulcer and it is thought to be associated with the development of gastric atrophy, intestinal metaplasia, and carcinoma[1,4], but the mechanism is not fully understood and the long-term effect of medical eradication not yet proven. A recent study[5] has shown that *H. pylori* has N-acetyltransferase activity and the researchers speculate that the bioactivation of food-borne heterocyclic aromatic amines into genotoxic and carcinogenic products in the stomach can be a promoter in the pathogenesis of gastric cancer.

The purpose of the present study was to determine the occurrence of *H. pylori* infection in persons with intellectual disability (ID) in residential care who presented with severe dyspeptic symptoms, and to monitor clinically the effect of treatment if *H. pylori* was found.

## METHODS

The Division for Mental Retardation under the Ministry of Labour and Social Affairs in Israel provides medical, social, and educational services to more than 6,000 persons with intellectual disability retardation in 53 residential care centers. One center in the central area of Israel was selected for this pilot study.

The study was initiated in January 1999 and it was decided to investigate every person for *H. pylori* who presented to the medical clinic of the center with severe dyspeptic symptoms. This method was used, instead of an examination of all residents, due to economic restraints by the Sick Fund (HMO) Laboratory on examinations.

Each person was examined clinically by the physician of the center, decisions made on further investigations or referrals, and a blood specimen drawn for IgG antibodies to *H. pylori* (examined at the Sick Fund Laboratory by ELISA, Pharmatop Millenia).

In the case of positive serology for *H. pylori* (IgG antibody level over 40 International Units), treatment was initiated, either as amoxycillin trihydrate 1,000 mg twice daily, metronidazole 1,000 mg twice daily and pantoprazole 40 mg twice daily (type 1) or omeprazole 20 mg twice daily (type 2) for 1 week. Medication in the center was given individually to each patient and the patient observed by the nurse while the medicine fully swallowed. If symptoms persisted omeprazole was continued for 1 month. Each patient was followed by the clinic for symptoms after the end of treatment and some patients followed by the gastroenterologist at the local specialist clinic.

## RESULTS

The center studied had 224 adult persons (63 females and 161 males), 100 persons between the ages of 19 and 45 years, 100 persons between 46 and 60 years, and 24 persons above 61 years; 70 persons had mild mental retardation and 154 had moderate mental retardation.

From January 1999 to May 2001 a total of 43 persons presented with severe dyspeptic symptoms and 42 persons (98%) were found to have *H. pylori* infection. Of these 42 persons (26 males, 16 females) the age range was 29–64 years with a mean of 45 years and the mean years of institutionalization, 20 years (range 6–39 years); 13 had mild and 29 moderate mental retardation. In the 21–30 year age group, 100% were positive for *H. pylori*. In the 31–50 year age group, 97% were positive and in those over 51 years, 100% positive. In the persons with mild mental retardation 93% were positive and 100% were positive in the group with moderate mental retardation. Thirty-two patients were treated with type 1 treatment, ten with type 2, and six patients were also treated with omeprazole for an extra month due to persistent symptoms.

At follow-up, clinically all patients had improvement and only six patients still had minor complains. Only eight were re-tested for IgG antibody level after treatment, six with over 50% reduction in *H. pylori* antibody titre, and two without change but with less clinical symptoms. A screening of all 42 persons after treatment was not possible due to restrictions by the Sick Fund (HMO) laboratory.

## DISCUSSION

Twenty years ago nobody had ever heard about *H. pylori* and, after an initial rejection by a skeptical scientific community, it has now been recognized as a major public health problem and one of the world's most common pathogens with a colonization of about 60% of the general population[6,7]. The mode of transmission of this pathogen is still not known, but the prevalence increases with age, disadvantaged socioeconomic conditions, and poor living standards although most individuals never develop clinical disease.

A recent review[8] of the prevalence of *H. pylori* in asymptomatic populations from Algeria, Israel, Saudi Arabia, and Turkey revealed that the prevalence is similar to findings from developing countries with a high level of infection in childhood (40-70%) and an age increase reaching 85-90%. Israel had a low prevalence among children (10%), but a rapid increase in the second decade of life to 39% and reaching 79% in persons over 60 years of age. An interesting finding was the higher prevalence rate in communal settlements (72%) compared to persons in urban dwellings (65%)[8,9]. The prevalence of *H. pylori* in patients with gastrointestinal symptoms and endoscopy performed was also reported from Egypt, Iran, Israel, Oman, Saudi Arabia, the United Arab Emirates, and Yemen[8]. Patients suffering from gastritis and peptic ulcer disease showed similar rates of infection as reported from Europe, the United States, and Africa, namely, 71–92%. Our study revealed a high rate of 98% in this group of symptomatic patients, but we were not able to make a prevalence study due to economic restraints on laboratory examinations.

Another study from Israel[10] investigated the prevalence of *H. pylori* in elderly nursing patients (aged 70 years or more) admitted to a medical center and found that the length of stay in the nursing home had a strong correlation. Patients with a short stay had a 63% prevalence, whereas patients with stays longer than 15 months had an 84% prevalence. The patients in our study had a mean institutionalized stay of 20 years, but since we were unable to test all individuals, such a comparison could not be made in our case.

Studies of *H. pylori* infection in the population of persons with mental retardation, developmental disability, or intellectual disability have not been performed before in Israel, but a few case stories and a few larger scale studies performed around the world.

Lubani et al.[11] from Kuwait described two siblings with developmental disability, cystic fibrosis and *Helicobacter* gastritis; Mauk[12] from Philadelphia described a 6-year-old boy with severe mental retardation, rumination, and several other behavioral disorders. *Helicobacter* infection was found on examination, treated and clinical improvement found as decreased frequency of rumination and gain of weight. Researchers from Italy[13] described a 14-year-old patient with microcephaly, ptosis, micrognathia, tetralogy of Fallot, mental retardation, *Helicobacter* infection, and deletion of chromosome 18, who developed gastric carcinoma.

In a study from Delaware[14], five children with profound neurologic impairment undergoing evaluation for gastrointestinal symptoms and subsequently in seven out of 61 children (prevalence of 11%) evaluated for *H. pylori* infection was identified as the cause of antral gastritis.

A study in 1995 from the United Kingdom[15] analyzed stored sera from 424 hospital residents with severe learning disabilities and compared it with 267 age and sex matched controls from the local non-residential population. The hospital residents under 40 years of age had an 87% prevalence of *H. pylori* compared with 24% for controls, whereas the overall prevalence for all ages was 87% for residents and 43% for controls.

Another larger study (338 intellectually disabled and 254 controls) from Holland[16] found a prevalence of 5% in children and 50% in the elderly in the general population, whereas 83% of the disabled and 27% of the healthy employees were infected. The presence of *Helicobacter* was significantly associated with male gender, longer duration of institutionalization, an IQ below 50, rumination, and a history of upper abdominal symptoms. For the employees the association was a higher level of physical contact with the disabled, longer duration of employment, and upper abdominal symptoms.

The mode of transmission of *H. pylori* infection is not clear. The bacteria can be cultured from feces, so fecal spread is one mode, but oral spread is also a possibility. A recent study from Switzerland among 92 gastroenterologists and 168 healthy controls revealed that gastroenterologists have a higher risk of aquiring *H. pylori* and would suggest oral infection via microscopic droplets of gastric juice produced by manipulating endoscopic instruments[17]. The studies of *H. pylori* infection in the population of persons with intellectual disability in residential care or elderly people living in nursing homes[18] is another opportunity to consider the mode of transmission. These two populations are known to live in a confined space, to take objects in the mouth and share food, to inflict injuries to self or others, to dribble or spit, to scratch and bite, to self-mutilate, and to have unhygienic toilet habits, thus suggesting several modes of transmission. The study from Holland[16] suggests a high incidence in this population and also transmission to care staff.

The rate of treatment success in our study (83%) was higher than the Delaware study[14], where only 50% of the patients had complete resolution of symptoms after treatment. The long-term treatment success should be studied as well in order to see the effect on the development of gastrointestinal cancer.

## CONCLUSION

It seems from several studies[15,16] that persons with mental retardation, developmental disability, or intellectual disability in residential care are at a higher risk for *H. pylori* infection, especially after some years in the institution. A study from Holland[16] also showed that staff exposed over time are prone to infection. On the basis of these studies and our study we recommend investigations for *H. pylori* in this population (by serology and not urea breath test, since that method is difficult in our population), when the patient develops decreased appetite, nausea, epigastric abdominal pain, or dyspepsia and, in case of positive serology, anti-*Helicobacter* triple drug treatment of 1 week duration should be implemented.
